# Infant feeding practices and maternal socio-demographic factors that influence practice of exclusive breastfeeding among mothers in Nnewi South-East Nigeria: a cross-sectional and analytical study

**DOI:** 10.1186/1746-4358-9-6

**Published:** 2014-05-20

**Authors:** Stanley Onah, Donatus Ignatius Chidiebere Osuorah, Joy Ebenebe, Clement Ezechukwu, Uchenna Ekwochi, Ifeyinwa Ndukwu

**Affiliations:** 1 Department of Paediatrics, Nnamdi Azikiwe University teaching Hospital, Nnewi, Anambra state, Nigeria; 2 Child Survival Unit, Medical Research Council Unit, Fajara, The Gambia; 3 Department of Paediatrics, Enugu State University Teaching Hospital, Enugu, Nigeria

**Keywords:** Exclusive breastfeeding, Infant feeding practice

## Abstract

**Background:**

Malnutrition is an underlying factor in more than 50% of the major cause of infant mortality-Pneumonia, diarrhoeal disease and measles which account for 70% of infant mortality. Therefore, programs to promote adequate nutrition for age can help reduce mortality from these disease conditions and indispensible to achievement of MDG 4.

**Aim:**

To describe the feeding practices of infants below six months of age and determine maternal socio-demographic factors that influences the practice of exclusive breastfeeding (EBF) among mothers in Nnewi, south-east Nigeria*.*

**Methods:**

Four hundred mother-infant pairs attending the infant welfare clinic of the Nnamdi Azikiwe University teaching hospital (NAUTH) during 2012 were consecutively recruited after meeting the study inclusion criteria. Data on breastfeeding were based on infant feeding practice in the previous 24 hours. Exclusive breastfeeding was defined as infant feeding with only breast milk.

**Results:**

Awareness (95.3%) and knowledge (82.0%) of EBF was high among surveyed mother but the practice of EBF (33.5%) was very low. Positive attitude towards EBF practice was shown by many (71.0%) of surveyed mothers. EBF practice decreased with increasing infant age, OR 0.72 (95% CI 0.34, 1.51) for 1–2 months, OR 0.58 (95% CI 0.23, 1.44) for 3–4 months and OR 0.20 (95% CI 0.06, 0.73) for 5–6 months compared to infants < 1 month old. Maternal education, socioeconomic class, mode of delivery and infants first feed were retained as important maternal predictors of EBF practice after adjustment for confounders. Decreased likelihood of EBF practice was found among mothers of lower educational attainment, OR 0.33 (95% CI 0.13, 0.81), mothers who delivered through caesarean section, OR 0.38 (95% CI 0.18, 0.84), mothers of higher socio-economic status [(middle class, OR 0.46 (95% CI 0.22, 0.99) and upper class, OR 0.32 (95% CI 0.14, 0.74)] while increased likelihood of EBF practice was seen in mothers who gave their infants breast milk as their first feed, OR 3.36 (95% CI 1.75, 6.66).

**Conclusion:**

Knowledge and awareness does not translate to practice of EBF. More effort by health workers and policy makers should be directed to mothers along the fault lines to encourage the practice of EBF.

## Background

Despite some improvements in child mortality rate in Africa, neonatal mortality has largely remained the same or worsened in many countries. In 2003, neonatal mortality accounted for almost 40 per cent of estimated 9.7 million children under-five deaths and for nearly 60 per cent of infant deaths. According to UNICEF in 2006 of the 10 million deaths in under-5 children recorded that year, 4 million die within the first month of life, half of these within the first 24 hours [1]. Because malnutrition increases a child’s risk of dying from many diseases — most prominently measles, pneumonia, and diarrhoea which are the highest cause (70%) of neonatal deaths — programs to improve nutrition can reduce mortality from several diseases simultaneously.

Efforts to promote modest nutritional improvements such as changes in feeding behaviour will have a beneficial impact on mortality rates over time. Feeding practices adopted by mothers depends on the knowledge, attitude, socio-cultural tradition they are exposed to [2]. Owing to the known nutritional and health benefits to the infant, the World Health Organization recommends that women in resource-poor countries exclusively breastfeed until their babies reach 6 months of age [3].

The Baby Friendly Hospital Initiative (BFHI) was introduced in Nigeria in 1992 to help educate and encourage breastfeeding practice among mothers. Some studies in Nigeria have shown that mothers who delivered in a health institution designated as baby friendly are more likely to practice exclusive breastfeeding (EBF) and breastfeed their infants for a longer time [4,5]. Another study in south-west Nigeria showed that mothers who had knowledge of BFHI but no contact with BFHI designated hospitals had significantly less incidence of practicing EBF compared to those who with knowledge and contact with a BFHI designated hospital. However a national survey done in 2008 showed that EBF rates still remains very low (13%) in Nigeria [6]. This is thought to be because of several factors associated with the mothers’ and the environments.

Several studies have documented the impact of cultural factors, maternal age, marital status, family income/social class, mode of delivery, time of initiation of first breastfeeding and proximity to babies on feeding pattern [7-10]. Outside maternal factors, studies have also shown that the babies’ general behaviour influence what feed they receive [11]. However none of these studies had explored in details the different feeding options and why mothers adopt the infant feeding practices they do. This study therefore tries to investigate the infant feeding practices, factors that influence the practice of EBF and reasons why mothers adopt these infant feeding methods in Nnewi in South-East Nigeria. The findings of this study will help inform policies targeted at behaviours among mothers that seek to enhance the practice of the lifesaving EBF in Nigeria and other developing countries where infant mortality rate is still unacceptably high.

## Methods

### Study area

This study was conducted in the Infant Welfare Clinic (IWC) of Nnamdi Azikiwe Teaching Hospital (NAUTH) Nnewi in 2012, which is a public medical institution in Anambra State, south-east Nigeria. Nnewi, the host town of NAUTH is located on latitude 6° 01´N and longitude 6° 55´E [12]. It is the second largest town in Anambra State, southeast Nigeria. It is an industrial hub of not only Anambra State but South-East Nigeria. The industries include: motor and motorcycle spare parts manufacture and merchandise, electrical cable manufacture and oil palm production. Majority of the inhabitants are Igbo by tribe and Christianity is by far the dominant religion.

NAUTH is the only tertiary health institution in Anambra State, South Eastern Nigeria providing health services to a catchment population of about 25,430,493 as at 2005 with an average annual growth rate of 3.4% [13]. It serves the entire state and also the neighbouring communities from the surrounding states of Enugu, Delta, Abia, Ebonyi, Kogi and Imo. The hospital maintains an Infant Welfare Clinic which runs three times in a week and is run by trained nursing staff. On average the centre attends to 30–40 mother-infant dyads on each clinic day. The clinic provides mainly free routine immunization services. Other services rendered include growth monitoring, and counseling in diverse aspects of child survival strategies.

### Study sample and selection process

This cross-sectional descriptive and analytical study carried out over a 6 months period involved apparently healthy infants under-six month with their mother and/or care-giver who attended the Infant Welfare Clinic of NAUTH Nnewi from August to December 2012. The mother-infant dyads that fulfilled the study inclusion criteria were consecutively enrolled.

The *inclusion criteria* includes: (i) Infants below six months of age who after history and physical examination were found not to have any acute or chronic systemic illness. (ii) Infants below six months of age whose mothers have given informed written consent. *Exclusion criteria*: (i) All infants who were HIV exposed, infected or whose HIV status were not known. (ii) All infants delivered prematurely. (iii) Low birth weight infants. (iv) Infants below six months of age who on history and physical examination were found to have organic or congenital abnormalities. (v) Infants below six months of age who had suffered from acute illness within the preceding two weeks. (vi) Infants whose mothers have died and (vii) Mother-infant dyads of multiple births i.e. twins, triplets etc. (viii) Infants below six months of age whose mothers refused giving consent.

### Data collection

Data collection was done by the 4 of the 6 principal researchers and two research assistants (interns who are qualified medical doctors). They were trained in the art of interviewing the mothers, desired clinical examination and the protocol of referral. The acceptable responses to the questions designated “interviewer to determine” were communicated to them just before commencement of data collection daily. The researchers often randomly went back to interview the mothers for quality control checks. These were all geared towards ensuring that study criteria were well applied. Before data collection mothers were assured that refusal to participate in the study will in no way affect the welfare services for their infants. Interview was done before receiving services in majority of the study participants because of the long queue and waiting time usually present in the infant welfare clinic.

The data collection tool was a pre-tested interviewer administered questionnaire which was completed by questioning the mother and taking measurements of the infants’ weight, length and head circumference [14]. Information was collected regarding parental place of origin and domicile, maternal age, marital status, educational attainment and occupation. The parental socio-economic class was derived based on Oyedeji’s classification [15]. Data were also collected about mothers’ antenatal clinic attendance, place of delivery, mode of delivery and time of delivery. The categorization of the specific infant feeding option practiced by the mother was based on mothers’ past 24 hour dietary recall [14]. Baby’s first feed, everything baby took in the previous 24 hours, reason for stopping breastfeeding etc. were documented. There was also a section that sought to find out whether the mothers ever heard of exclusive breastfeeding, mothers’ knowledge of the meaning, willingness to practice it if given the opportunity and reason for rejecting exclusive breastfeeding (where applicable).

Prior to enrolment of any mother-infant dyad, specific enquiries on the elements of the exclusion criteria were made including the HIV status. HIV status of mother-infant dyad whose birth was registered in NAUTH was obtained from the hospital records for mothers who gave consent to participate in the study. For mothers who gave birth outside the hospital without documented evidence on their HIV status, this information was obtained by mothers recalling the results of HIV if done during pregnancy. For mothers who did not do the HIV testing during pregnancy of index infant or those who cannot recall if HIV was done during pregnancy or those who were cannot recall the result of the HIV screen or those not sure of their status, were all excluded from the study.

For the infants, information on the sex, birth rank, time of first intake of feed and/or water, reasons for delay beyond 30 minutes, first feed given to baby, colostrums intake, etc. were sought from the mothers.

### Independent variables

Maternal age in years categorised as 25 or less, 26–30, 31–35, and ≥36; educational level of mothers categorised as no education, primary education, secondary and tertiary education; marital status was categorised as not married and currently married, occupation of mothers during previous 6 months after birth was categorised as none, unskilled, semi-skilled and skilled; distance of home from work place was grouped as within residence, nearby (less than 30 minutes walk) and farther way (more than 30 minutes walk); socioeconomic class of mother was categorised as; lower, middle, rich [15]. Parity of mother was categorised as 1, 2–3, and ≥4; maternal leave entitlement for working mothers was categorised as no and yes; mothers who had maternity leave were further categorised based on duration of the leave as 1–2, 3–4 and 5–6 months; use of antenatal care was categorised as no or yes; mode of delivery was classified into vaginal and caesarean section. Infant’s age was categorised as < 1 months, 1–2 months, 3–4 months and 5–6 months; gender of the infant grouped as male or female; and birth rank grouped into first, second, third, fourth and fifth or higher.

### Outcome variable

The outcome variable is the proportion of mothers who exclusively breastfed (i.e. practised EBF) their infants aged 0–6 months during the previous 24 hours [16]. This was categorized into (i) Yes for mothers who practised exclusive breastfeeding and (ii) No for mother that did not practice exclusive breastfeeding. Table 1 below shows WHO definition of the different feeding options considered in this study [17,18].

**Table 1:** WHO definition of selected feeding practices

**Infant-feeding (IF) option**	**What this type of IF requires infants to receive**	**What this type of IF allows infants to receive**	**What this type of IF does not allow infants to receive**
Exclusive breastfeeding **(EBF)**	Breast milk (including milk expressed or from a wet nurse)	Oral rehydration solution (ORS), Drops or syrups (vitamins, minerals, medicines)	Anything else
Predominant breastfeeding **(PBF)**	Breast milk (including milk expressed or from a wet nurse) as the predominant source of nourishment	Liquids (water and water based drinks, fruit juices, ORS), ritual fluids and drops or syrups (vitamins, minerals, medicines)	Anything else (in particular, non-human milk, food-based fluids)
Breastfeeding with complementary foods **(CBF)**	Breast milk (including milk expressed or from a wet nurse) and solid or semi solid foods	Anything else: any food or liquid including non-human milk and formula	No restrictions applicable
Breastfeeding **(BF)**	Breast milk (including milk expressed or from a wet nurse)	Anything else: any food or liquid including non-human milk and formula	No restrictions applicable

### Data analysis

The Predictive Analytics Software (PASW) Statistic 19.0 statistical package was used for data analysis. The Pearson chi-square (*χ*^2^) test was used to study the differences between the independent variables and EBF practice. Binary logistic regression was also used for analysis of maternal variables, in order to examine their individual effects on EBF practice. Multiple binary logistic regression was used for multivariate analysis, to determine the main maternal factors which predicted EBF practice in this population, when all other maternal factors in this study were adjusted for. All predictors were entered in the same block. The largest category in each predictor variable was used as the reference category. For all statistical tests performed, it was ensured that the assumptions for carrying out these specific tests were met. Statistical significance was set at P-value < 0.05 and 95% Confidence Interval (95% CI) was used. Results are presented using percentages, Odds Ratios and 95% CIs where appropriate.

### Ethical consideration

Ethics approval was obtained from the Nnamdi Azikiwe University Teaching Hospital Ethics Committee (NAUTHEC) with reference number NAUTH/CS/66/VOL3/50. Informed consent (written) was obtained from every mother in her own right and on behalf of her child before recruitment. Participation in the study was entirely voluntary and no financial inducement whatsoever was involved. Voluntary withdrawal at any stage of interaction was guaranteed for all subjects without any adverse effect for the mother or the baby. All information was handled with strict confidentiality.

## Results

### Characteristics of surveyed mother-infant dyads

**Figure 1:**
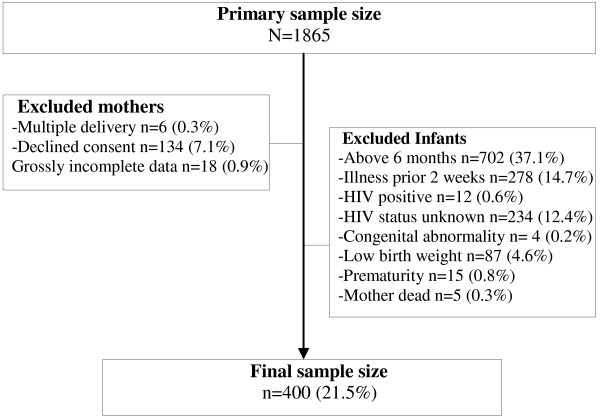
Selection procedure.

From a primary sample of 1865 mother-infant dyads seen during the period of study, 534 (28.6%) were found eligible having met the set study criteria. One hundred and thirty- four (134) declined participating in the study. This gives a recruitment fraction of 0.75 (400/534) and a final sample size of 400 mother-infant dyads (see Figure 1). Most of the mothers surveyed were from the lower socioeconomic class and had secondary school education as their highest educational attainment (see Table 2). This is very representative of the clientele in the study area as poorer families due to the cheaper service fee in NAUTH compared to private hospital patronizes the teaching hospital relatively more. Majority 225 (56.2%) of infants included in the survey were between 1–2 months compared to 53 (13.3%), 76 (19.0%) and 46 (11.5%) within the < 1, 3–4 and 5–6 months age bracket respectively.

**Table 2:** Infant feeding methods of surveyed mothers

**Variables**		**Infant feeding practice n (%)**			
	**Total**	**Exclusive (EBF)**	**Predominant (PBF)**	**Complementary (CBF)**	***p***
**Age of infants (months)**	**N = 400**	**N = 134**	**N = 111**	**N = 155**	
Less than 1	53 (13.3)	22 (41.5)	27 (50.9)	4 (7.6)	**0.01**
1-2	225 (56.2)	84 (37.3)	70 (31.1)	71 (31.6)	0.20
3-4	76 (19.0)	23 (30.3)	13 (17.1)	40 (52.6)	0.05
5-6	46 (11.5)	5 (10.9)	1 (2.1)	40 (87.0)	**0.01**
**Age of mother (years)**	**N = 398**	**N = 134**	**N = 110**	**N = 154**	
25 or less	106 (26.6)	34 (32.1)	27 (25.5)	45 (42.6)	0.78
26-30	159 (40.0)	59 (37.1)	51 (32.1)	49 (30.8)	0.21
31-35	98 (24.6)	32 (32.7)	26 (26.5)	40 (40.8)	0.93
36 or more	35 (8.8)	9 (25.7)	6 (17.1)	20 (57.2)	0.10
**Educational level of mother†**^**1**^	**N = 396**	**N = 134**	**N = 111**	**N = 151**	
Primary	89 (22.3)	18 (20.2)	26 (29.2)	45 (50.6)	**0.03**
Secondary	170 (42.5)	55 (32.4)	52 (30.6)	63 (37.0)	0.86
Tertiary	137 (34.3)	61 (44.5)	33 (24.1)	43(31.4)	0.08
**Socioeconomic class**	**N = 400**	**N = 134**	**N = 111**	**N = 155**	
Lower	185 (46.3)	48 (26.0)	57 (30.8)	80 (43.2)	0.19
Middle	150 (37.5)	55 (36.7)	42 (28.0)	53 (35.3)	0.72
Upper	65 (16.2)	31 (47.7)	12 (18.5)	22 (33.8)	0.07

†^1^- Missing values (mothers with no education (4) were excluded.

### Infant feeding practice

Three hundred and eighty-two 382 (95.3%) of the 400 mothers have heard of exclusive breastfeeding (EBF). When asked to explain what it means 328 (82.0%) and 54 (13.5%) of the 382 mothers correctly and incorrectly respectively explained what EBF meant. Eighteen 18 (4.5%) of the 382 mothers did not respond.

Seventy nine 79 (19.8%) of mothers surveyed commenced feeding of their newborn within 30 minutes following birth, 49 (12.3%) within 1 hour, 115 (28.8%) and 114 (28.5%), 1–6 and beyond 6 hours respectively after birth. Forty-three, 43 (10.8%) of them could not remember when feeding was started for their newborn. Of the 278 (69.5%) who started feeds beyond the recommended 30 minutes after birth the following reasons was given for the delay in initiating breast milk for their newborns; Baby was not crying (hungry) 79 (28.9%), baby was too weak 13 (4.7%), not lactating 41 (14.8%), was too weak after delivery 53 (19.1%), was not asked to feed newborn 15 (5.4%), could not because delivery was through operation 50 (17.9%), maternal illness 4 (1.4%) and no reason 23 (8.2%).

A hundred and ninety-four 194 (48.4%) of the surveyed mother gave breast milk as the first feed to their newborns, plain water was given in 89 (22.3%) cases, infant formula in 67 (16.8%), glucose water in 48 (12.0%) and coconut water was first feed in 2 (0.5%) of cases. Reasons breast milk was not given as first feed seen in this study included breast milk not flowing 89 (33.5%), not strong enough to breastfeed 74 (27.8%), relatives fed baby without mothers notice 32 (12.0%), advice from relatives 10 (3.8%) and no reason 61 (22.9%). There were combinations of these reasons.

Exclusive breastfeeding was being practiced by 134 (33.5%) of the surveyed mothers, predominant breastfeeding by 111 (27.8%), and complementary breastfeeding by 155 (38.8%) (p = 0.002). Choice of feeding type was informed by advice from health workers in 30 (7.5%) mothers, advice from friends and/or relatives 88 (22.0%), adverts from prints and/or electronic media 10 (2.5%), cost factors 32 (8.0%), time factor 75 (18.8%), personal convenience 62 (15.5%) and no reason 103 (25.8%). There were also combinations of reasons.

Table 2 shows the infant feeding options adopted by mothers surveyed. For the 266 (66.5%) who did not practice EBF various reasons like baby cries too much 73 (27.7%), not gaining weight 17 (6.4%), pressure from relatives 41 (15.5%), work/business demands 23 (8.7%), felt baby was thirsty and needed water 101 (25.3%) and baby eats too much 9 (3.4%) were given for not practicing EBF. Only 284 (71.0%) of the surveyed mothers believed that EBF is the most ideal infant feeding option and agreed they would practice EBF if given the time and opportunity to do so. Conversely 87 (21.8%) did not share this opinion and would not practice EBF even when conditions are right. Twelve, 12 (13.8%) of these 87 mothers were concerned about getting too fat as EBF makes them eat too much, 22 (25.3%) said the demand of EBF was too much, 13 (15.0%) feared the baby will refuse other feed if EBF making weaning difficult, while 12 (13.8%) felt that water is essential to life so giving water is the right thing to do. Different combinations of these reasons were also given. Finally amongst mother who were not practicing EBF, majority 65 (36.5%) introduced water or other feed when their child was less than 1 month old, 27 (15.3%) on or after 1 month, 16 (9.1%) on or after 2 months, 27 (15.4%), 15 (8.5%) and 5 (2.9%) on or after 3, 4 and 5 months of age respectively. Table 3 shows maternal and infant’s factors associated with practice of exclusive breastfeeding.

**Table 3:** Cross tabulation of maternal and infants factors and practice of EBF

**Variables**	**N**	**Exclusive breastfeeding (%)**		***chiχ***^**2**^
	**n (%)**	**No**	**Yes**	***p***
*Maternal variables*				
**Age of mother (years)**	**N = 398**	**N = 264**	**N = 134**	2.00
25 or less	106 (26.6)	72 (67.9)	34 (32.1)	0.57
26-30	159 (40.0)	100 (62.8)	59 (37.2)	
31-35	98 (24.6)	66 (67.3)	32 (32.7)	
36 or more	35 (8.8)	26 (74.2)	9 (25.8)	
**Educational level of mother**	**N = 396**	**N = 262**	**N = 134**	14.52
Primary	89 (22.3)	71 (79.7)	18 (20.3)	**0.01**
Secondary	170 (42.5)	115 (67.6)	55 (32.4)	
Tertiary	137 (34.3)	76 (55.4)	61 (44.6)	
**Socioeconomic class**	**N = 400**	**N = 266**	**N = 134**	11.29
Lower	185 (46.3)	137 (74.0)	48 (26.0)	**0.01**
Middle	150 (37.5)	95 (63.3)	55 (36.7)	
Upper	65 (16.2)	34 (52.3)	31 (47.7)	
**Occupation of mother**	**N = 400**	**N = 266**	**N = 134**	12.10
None	97 (24.2)	60 (61.8)	37 (38.2)	**0.01**
Unskilled	140 (35.0)	109 (77.8)	31 (22.2)	
Semi-skilled	86 (21.5)	52 (60.4)	34 (39.6)	
Skilled	77(19.3)	45 (58.4)	32 (41.6)	
**Distance of office from home**	**N = 303**	**N = 206**	**N = 97**	3.19
Within residence	59 (19.5)	42 (71.2)	18 (28.8)	0.20
Nearby (<30 minutes walk)	154 (50.8)	109 (70.8)	44 (29.2)	
Farther (>30 minutes walk)	90 (29.7)	55 (61.2)	35 (38.8)	
**Maternity leave**	**N = 288**	**N = 195**	**N = 93**	2.11
No	154 (53.5)	110 (71.4)	44 (28.6)	0.15
Yes	134 (46.5)	85 (63.5)	49 (36.5)	
**Antenatal care**	**N = 400**	**N = 266**	**N = 134**	3.40
No	32 (8.0)	26 (81.2)	6 (18.8)	0.07
Yes	368 (92.0)	240 (65.2)	128 (34.8)	
**Mode of delivery**	**N = 400**	**N = 266**	**N = 134**	15.36
Vaginal (spontaneous or assisted)	320 (80.0)	198 (61.8)	122 (38.3)	**0.01**
Caesarean section (CS)	80 (20.0)	68 (85.0)	12 (15.0)	
**Time from delivery to first feed**	**N = 357**	**N = 232**	**N = 125**	7.24
Within 1 hr	128 (35.9)	80 (62.5)	48 (37.6)	**0.03**
1-6 hrs	115 (32.2)	67 (58.2)	48 (41.7)	
Greater than 6 hrs	114 (31.9)	85 (74.5)	29 (25.5)	
*Infant variables*				
**Age of infants (months)**	**N = 400**	**N = 266**	**N = 134**	13.94
Less than 1	53 (13.3)	31 (58.4)	22 (41.6)	**0.01**
1-2	225 (56.2)	141 (62.6)	84 (37.4)	
3-4	76 (19.0)	53 (69.7)	23 (30.3)	
5-6	46 (11.5)	41 (89.1)	5 (10.9)	
**Sex**	**N = 400**	**N = 266**	**N = 134**	0.33
Male	211 (52.8)	143 (67.7)	68 (32.3)	0.57
Female	189 (47.2)	123 (65.0)	66 (35.0)	
**Birth rank**	**N = 400**	**N = 266**	**N = 134**	
First	126 (31.5)	92 (73.0)	34 (27.0)	6.10
Second	80 (20.0)	49 (61.2)	31 (38.8)	0.19
Third	73 (18.2)	47 (64.3)	26 (35.7)	
Fourth	62 (15.5)	36 (58.0)	26 (42.0)	
Fifth and higher	59 (14.8)	42 (71.1)	17 (28.9)	
**Colostrum given**	**N = 400**	**N = 266**	**N = 134**	7.78
No	46 (11.5)	39 (84.8)	7 (15.2)	**0.01**
Yes	354 (88.5)	227 (64.1)	127 (35.9)	
**Baby’s first feed**	**N = 400**	**N = 266**	**N = 134**	
Water and/or water based solutions	139 (34.8)	105 (75.5)	34 (24.5)	32.86
Breast milk	194 (48.4)	103 (53.1)	91 (46.9)	**0.01**
Formula based- milks	67 (16.8)	58 (86.7)	9 (13.3)	

### Maternal and child factors influencing practice of EBF

Table 4 shows analysis of maternal and child variables and how they influence the practice of EBF. In the final regression model, educational status, socioeconomic class, mode of delivery, age of the child and first feed given to neonate after birth were retained as important determinants of EBF practice (see Table 4). Mothers with primary education OR 0.33 (95% CI 0.13, 0.81) and those with secondary education OR 0.57 (95% CI 0.29, 1.11) were 0.33 and 0.57 times less likely to practice EBF compared to mothers with tertiary education. Mothers who delivered through caesarean section were 0.38 times less likely to exclusively breastfeed their newborn OR 0.38 (95% CI 0.18, 0.84) compared to those who delivered vaginally. In others words mothers who had non operational deliveries were 2.6 times more probable to practice EBF than those who delivered through operation. Also, EBF practice decreased with increasing infant age, OR 0.72 (95% CI 0.34, 1.51) for 1–2 months, OR 0.58 (95% CI 0.23, 1.44) for 3–4 months and OR 0.20 (95% CI 0.06, 0.73) for 5–6 months compared to infants less than 1 month old.

**Table 4:** Logistic analysis of maternal and infants factors associated with the practice of EBF

**Variables**	**Crude OR (95% CI)**	**Adjusted OR (95% CI)**
**Age of mother (years)**		
25 or less	1	-
26-30	1.25 (0.74, 2.10)	-
31-35	1.03 (0.57, 1.85)	-
36 or older	0.73 (0.31, 1.73)	-
**Mother educational level**		
Primary	0.32 (0.17, 0.59)	0.33 (0.13, 0.81)
Secondary	0.59 (0.37, 0.95)	0.57 (0.29, 1.11)
Tertiary	1	1
**Socioeconomic class**		
Lower	1	1
Middle	1.65 (1.45, 4.68)	0.46 (0.22, 0.99)
Upper	2.60 (1.04, 2.64)	0.32 (0.14, 0.74)
**Occupation of mother**		
None	1	1
Unskilled	0.46 (0.26, 0.82)	0.22 (0.01, 3.85)
Semi-skilled	1.06 (0.56, 1.92)	0.45 (0.03, 8.34)
Skilled	1.15 (0.63, 2.12)	0.64 (0.04, 11.9)
**Distance of workplace from home**		
Within residence	0.61 (0.31, 1.25)	-
Nearby (<30 minutes walk)	0.63 (0.37, 1.09)	-
Farther (>30 minutes walk)	1	-
**Maternity leave**		
No	1	-
Yes	1.44 (0.88, 2.37)	-
**Antenatal care**		
No	0.43 (0.17, 1.08)	-
Yes	1	-
**Mode of delivery**		
Vaginal (spontaneous or assisted)	1	-
Caesarean section (CS)	0.29 (0.15, 0.55)	-
**Age of child (months)**		
Less than 1	1	1
1-2	0.84 (0.46, 1.54)	0.72 (0.34, 1.51)
3-4	0.61 (0.29, 1.27)	0.58 (0.23, 1.44)
5-6	0.17 (0.06, 0.51)	0.20 (0.06, 0.73)
**Sex**		
Male	1	-
Female	1.28 (0.75, 1.71)	-
**Birth rank**		
First	1	-
Second	1.71 (0.94, 3.11)	-
Third	1.49 (0.81, 2.78)	-
Fourth	1.95 (0.98, 3.71)	-
Fifth and higher	1.10 (0.55, 2.18)	-
**Colostrum given**		
No	1	1
Yes	3.12 (1.36, 7.17)	0.60 (0.22, 1.64)
**Infants first feed**		
Water and/or water based solutions	1	1
Breast milk	2.73 (1.69, 4.40)	3.36 (1.75, 6.66)
Formula based- milks	0.48 (0.22, 1.07)	0.54 (0.18, 1.63)

Lastly mothers who gave their infant’s breast milk as the first feed were 3.36 times more likely to practice exclusive breastfeeding than mothers who gave water and/or water based solutions as first feed to their infants, OR 3.36 (95% CI 1.75, 6.66) while those that gave infant formula were 0.54 times less likely to practice exclusive breastfeeding compared to mothers who gave water and/or water based solutions to their infants as its first feed, OR 0.54 (95% 0.18, 1.63). In explicit terms, mothers who gave breast milk as the first feed to their newborn were 3.36 and 6.22 times more likely to practice EBF compared to mothers who gave water/water based solution and infant formula respectively as first feed to their newborns.

## Discussion

The awareness and correct knowledge of EBF concept was very high but its practice was distinctly lagging. This mismatch between knowledge and practice has also been reported in another study in the same south eastern Nigeria [19]. It was evident from this study that awareness and knowledge do not equate to practice. Inferentially, our mothers, probably, have not come to accept or understand the critical vital benefit of EBF or that the challenges to its practice are deemed insurmountable for now. To overcome this, emphasis should be shifted from mere dissemination of information on EBF to empirically helping mothers resolve potential challenges highlighted in this study.

On average only about 34% of the babies were on EBF and this proportion was seen to rapidly decline from about 64% at 1–2 month to around 4% at the 5–6 months. As much as this trend has been variously reported [20,21], the rate was much lower than in Port Harcourt Southern Nigeria [20] and Sokoto in Northern Nigeria [22] which were 58% and approximately 41% respectively at 6 months. Differences in study design might have accounted for this wide variation in rates. The Port Harcourt and Sokoto studies were both longitudinal and interventional studies and since active mobilization and monitoring have been documented to positively impact EBF practices, the reported higher rates in these locations could be attributed to these interventions. This study being of a cross-sectional design would have been devoid of such influence. The unacceptably low rate of EBF seen in this study makes it imperative that attitude-leveraging measures should be found and implemented to optimize the benefits from proper infant feeding practices.

Complementary breastfeeding which involves use of both breast milk, infant formula and other non milk feeds was practiced by significantly more (39%) mothers surveyed compared to EBF (34%) and predominant breastfeeding (28%). Not surprisingly stratification analysis showed that mothers whose infants were older and mothers with lower education attainment practiced complementary breastfeeding than other infant feeding option. The reason for these findings is easily explained. Most mothers usually start introducing other types of feeds as child gets older and able to tolerate these feeds in order to give them (mothers) time to attend to other activities. Likewise mothers with higher education will more likely understand and be better informed of the benefits of EBF thus delay introduction of other feeds compared to mothers with lower educational attainment. This study clearly showed that mothers with tertiary education were more likely to practice EBF compared to those with secondary and primary education. This was similar to the findings of Lawoyin et al. [9] in a study at Ibadan, Southwest Nigeria. With greater education, mothers are more likely to be abreast of the overriding benefits of EBF and therefore will be more motivated to practice it. Little wonder why maternal education has been long recognized as one of the child survival strategies adopted by UNICEF in its GOBIFF strategy [23].

This study observed that socioeconomic status had inverse association with EBF practice. Higher socioeconomic status was associated with lessened rate of EBF practice. This may be related to the notion of use of infant formula as a status symbol. One could also speculate that these mothers in the higher socioeconomic class, who are richer (sometimes by virtue of husbands wealth or families they are married into) but not necessarily better educated are able to afford and sustain infant formulas which are exorbitant in price. Furthermore, the occupation of mothers in this socioeconomic class would most likely interfere with the practice of EBF. This class of mothers is also more likely to travel for business engagements thereby hampering to some extent the preconditions for EBF practice. They may need to be better educated on the skill of expression of breast milk which their babies will be fed while they are unavoidably away.

Mode of delivery also had a strong influence on EBF Practice. Delivery by caesarean section was associated with less EBF practice. A study in Taiwan made similar observations [24]. This will not be unrelated to the limiting co-morbidities associated with such procedure. Such mothers would usually take long to recover from anaesthesia before thinking of recommended infant feeding practice. Also the issue of increased maternal stress following operative deliveries could delay onset time for lactation [25]. This finding was closely linked to the babies’ first feed. The use of prelacteal feeds which also was significantly associated with reduced EBF practice was common place amongst mothers that delivered by Caesarean section. It probably feels natural for them to continue what they had started the baby on when it seemed to them they had plausible excuse. This association between prelacteal feeds and non-EBF practice has also been reported from other studies [26,27]. It was instructive that even amongst mothers with normal vaginal delivery; use of prelacteal feeds was inversely associated with the practice of EBF. To reduce this detracting influence on EBF practice, measures should be taken to reduce deliveries by non-vaginal routes and also adopt protocols that minimize Caesarean pain and other co-morbidities when inevitably indicated. This way, the temptation to use prelacteal feeds and subsequent non-EBF practice will be lessened.

### Limitations

Breastfeeding practice information could only be acquired from the questions concerning a 24 hour–recall infant feeding practice. Several misclassifications might have occurred since a mother who has been exclusively breastfeeding her infant but for some reasons gave her infant water or some other liquids the previous day is considered not exclusively breastfeeding. Also, a mother who usually gave her infant water or some other complementary foods and for some reasons did not give her infant any the previous day will be considered exclusively breastfeeding. More so, during data collection, recall bias (especially on sensitive information) and mis-reporting of information by the participants and interviewers respectively may possibly affect data collected leading to error in data, analysis and interpretation. It is therefore recommended that the findings of this study be interpreted with caution in the light of these limitations.

## Conclusions

Exclusive breastfeeding practice is poor from this study but the awareness is remarkably high depicting significant knowledge-practice discordance. The current approach to EBF promotion in NAUTH being a Breastfeeding Friendly Hospital has at best only increased awareness without a corresponding improvement in its practice. Factors such as low maternal education, higher socioeconomic status, non-vaginal birth and use of prelacteal feeds were significant predictors of lower EBF practice. To optimally reap the potential benefits of proper infant feeding, effort needs to be shifted to strategies that not only sustains and promotes knowledge to those that confer behavioural change. Exclusive breastfeeding support groups may be able to serve this need.

Further multi-level analysis to understand the influence of other extraneous non-maternal factors like spousal factors, household and community and cultural practices/beliefs on EBF practice is suggested in order to guide policy makers and public health organizations in planning appropriate and adequate interventions to improving EBF practice.

## Competing interests

The authors declare that they have no competing interests. This study was completely sponsored by the authors.

## Authors’ contribution

SO, JE, and CE conceived and designed the study. SO, UE and IN were responsible for supervision of data collection and quality control. DICO analyzed the data, wrote the result and the first draft of the manuscript. SO, DICO and UE contributed to discussion, editing, and approved the text. JE and CE reviewed the final manuscript and supervised the work. All authors read and approved the final manuscript.
